# A survey on depression among infertile women in Ghana

**DOI:** 10.1186/1472-6874-14-42

**Published:** 2014-03-10

**Authors:** Abass Alhassan, Abdul Razak Ziblim, Sirina Muntaka

**Affiliations:** 1 Department of Human Biology, School of Medicine and Health Science, University for Development Studies, Tamale, Ghana; 2 Department of Nursing and Community health, School of Medicine and Health Science, University for Development Studies, Tamale, Ghana; 3 Department of Immunology, University of Nottingham/Queen’s Medical Centre, NG7 2UH England, UK

## Abstract

**Background:**

The desire of many young women to become parents may be influenced by the premium placed on children by society. In Africa, children are highly valued for social, cultural and economic reasons. Infertile and childless women in Africa are therefore confronted with a series of societal discrimination and stigmatization which may lead to psychological disorders such as anxiety and depression. Even though some research has been done on the prevalence of infertility in Ghana, very little is known about the psychological impact of childlessness among infertile women. The present study aimed to examine prevalence and severity of depression in relation to age, type of infertility and duration of infertility in Ghanaian infertile women.

**Methods:**

A total of 100 infertile women who met the selection criteria and had agreed to participate in the study were interviewed using the Beck Depression Inventory questionnaire from December 2012 to April 2013 at the Tamale Teaching Hospital, Tamale/Ghana. Data concerning socio-demographic characteristics such as age, monthly income, duration of infertility, marital status, educational level, number of previous conception, number of previous children, religion, as well as occupation of the respondents were recorded.

**Results:**

The prevalence of depression among the women was 62.0% with the level of depression showing a significant positive correlation with age of the women and the duration of infertility. The level of depression was significantly higher among subjects with low or no formal education and among the unemployed. Women with primary infertility also presented with high depression scores as measured by BDI.

**Conclusions:**

In conclusion, the prevalence of depression among the infertile women is high, especially among infertile women age 26 and above, those who are less educated, those with primary infertility, as well as those who have been diagnosed as infertile for more than 3 years. Interventions to decrease and prevent the development of severe depression among these patients should be considered.

## Background

Infertility as defined by WHO and others is the inability of a sexually active non-contraceptive using, non-lactating woman to have a live birth after 12 or more months of regular sexual intercourse [1,2]. The type and prevalence of infertility varies widely from one country to the other. In Sub-Saharan Africa, secondary infertility is the most prevalent type of infertility [3,4]. Secondary infertility is defined as the inability of a sexually active non-contraceptive using woman who has previously had a live birth to have a child despite cohabitation and the wish to become pregnant for at least 12 months [2,5-7]. The inability to have children is undeniably a very distressing experience in both men and women which can lead to major psychological disorders such as depression. Depression is said to be a major problem associated with infertility especially in Africa where children are highly valued for socio-cultural and economic reasons [6,8-10]. Childlessness often creates enormous problems for couples; especially for the women who are generally blamed for the infertility status of couples [11,12]. Infertility is of particular concern in Africa and Ghana in particular because of the extent of the problem and social stigma attached to it. Motherhood in Ghana is often the only way for women to enhance their status within their family and community. In some communities, the stigma of childlessness is so great that infertile women are socially isolated and neglected [13]. Studies from communities in Egypt [8], Nigeria [14], Mozambique [15] and the Gambia [16] showed that infertile women are often excluded from social events and ceremonies or may even be despised and perceived as inauspicious. Available evidence suggests that the social and psychological consequences of infertility are particularly profound for African women as compared to men [8,17].

Depression as a psychological consequences of infertility may play a significant role in the life of an infertile person and could subsequently affect the mutual relationship and the quality of life of a couple [18]. In some parts of Africa and elsewhere studies have demonstrated the psycho-social impact of infertility among infertile women [19]. In Ghana however, little is known about the prevalence and extent of depression among infertile women as well as factors that may predispose infertile women to depression. The aim of the present study was to determine the prevalence and severity of depression among Ghanaian infertile women in relation to duration of infertility and other socio-demographic characteristics in a cosmopolitan Ghanaian setting.

## Methods

This cross-sectional study was conducted from December 2012 to April 2013, involving 100 infertile women who were purposively recruited among patients attending the fertility clinic at the Obstetrics and Gynaecology unit of the Tamale Teaching Hospital in Ghana. After approval by the Ethics committee of the Hospital, informed consent of the respondents was obtained after explaining the purpose of the study to them. In order to be included in the study, respondents should have met the classical definition of infertility defined by the WHO as the inability of a sexual active non-contraceptive using woman to have a live birth after 12 or more months of regular sexual intercourse without a male factor. Women who had male factor infertility were excluded. In this study live birth was used as a measure of proven fertility (Because couples desire children, not simply pregnancies, infertility affects couples regardless of whether the aetiology lies in conception or in the progression of the pregnancy). Primary infertility as used in the present study refers to the inability of a sexually active non-contraceptive using and non-lactating woman to have a live birth after 12 or more months of regular sexual intercourse and secondary infertility refers to situation where a couple who has previously had a live birth but is subsequently unable to have one despite cohabitation and the wish to become pregnant for at least 12 months or more months.

Socio-demographic information including age, duration of infertility, marital status, educational level, type of marriage, number of previous children and religion were obtained from the respondents. The occupation, monthly income, whether the subjects were presenting with primary or secondary infertility were also obtained. Data relating to psychological impact of infertility was obtained using the Beck Depression Inventories (BDI).

### Beck depression inventory (BDI)

The test used was a modified and validated Persian version of Beck’s depression Inventory [20,21]. A full 21-items BDI was administered. This scale is a widely used measure for intensity of depression. Each item describes a specific behavioural manifestation of depression. Scores on each item can range from 0, indicating no depressive symptomatology, to 3, indicating a severe level of symptomatology. Total scale scores can thus range from 0 to 63. Scores of 17 or above indicates a clinically significant depression. The classification of depression scores involves:

1. 0–16 (without depression)

2. 17–27 (mild depression)

3. 28–34 (moderate depression)

4. 35–63 (severe depression).

### Statistical analysis

The relationship between categorical responses and explanatory variables were evaluated using chi-square test. Frequency tables were used for the descriptive purposes. One way analysis of variance was performed to find out significant difference between infertility duration on depression score. In all statistical tests, a value of P < 0.05 was considered significant. Data were analyzed using GraphPad Prism version 6.00 for Windows, (GraphPad Software, La Jolla California USA, http://www.graphpad.com).

## Results

### Socio-demographic characteristics

The mean age of the subjects was 30.5 years (SD = 6.3). Majority of the subjects 48.0% (48/100) were within 20-30 years age group followed by those in the 31-35 year group (32.0%) as shown in Table 1. About 78.0% of the women were self-employed engaging in petty trading and dress making with only 8.0% of them being employed as civil servants in the formal sector. A significant number of them (14.0%) were unemployed as shown in Table 1. Over 80.0% of the study subjects practice the Islamic faith with only 16.0% belonging to the Christian faith as indicated in Table 1. With respect to income level, majority (60.0%) were low income earners (Table 1). In all 54.0% of the study subjects had not attained any form of formal education with only 46% attaining at least basic formal education as indicated in Table 2. Majority of the women presented with secondary infertility (62.0%) with 38.0% presenting with primary infertility (Table 2).

**Table 1:** Socio-demographic characteristics of the study population

**Parameter**	**n**	**%**
**Age**		
20-25	24	24.0
26-30	24	24.0
31-35	32	32.0
36-40	16	16.0
>40	4	4.0
**Occupation**		
Unemployed	14	14.0
Self employed	78	78.0
Civil servant	8	8.0
**Religion**		
Islam	84	84.0
Christianity	16	16.0
**Income**		
Low	60	60.0
Middle	34	34.0
High	6	6.0

n = number of patients.

**Table 2:** Level of depression stratified by education, type of infertility and conception status

**Variable**	**n (%)**	**Level of depression**			
**Education**		**No depression (n)**	**Mild (n)**	**Moderate (n)**	**P value**
No Education	54 (54.0)	16	24	14	
Low Education	38 (38.0)	18	12	8	
High Education	8 (8.0)	4	4	0	
**Total**	**100 (100.0)**	**38**	**40**	**22**	**0.242**
**Type of infertility**					
Primary	38(38.0)	8	16	14	
Secondary	62(62.0)	30	24	8	0.004
**Conception**					
Ever conceive	58(58.0)	30	16	12	
Never conceive	42(42.0)	10	8	24	0.001
**Type of marriage**					
Monogamy	52 (52.0)	20	20	12	
Polygamy	48 (48.0)	20	12	16	0.299

### Depression among the study subjects

The results of the BDI showed that 62.0% of the subjects had some form of depression with only 38.0% of them showing no symptoms of depression. Analysis of the level of severity of depression revealed that a significant 40.0% and 22.0% of the subjects were suffering from mild and moderate depression respectively. None of the women had severe form of depression as shown in Table 2. The mean score of depression by educational level showed that women with no formal education tended to be more depressed than those who at least had some form of education with the level of depression decreasing with higher education even though this trend was not statistically significant (0.242) as shown in Table 2. The type of infertility presented by the women and previous conception both had significant effect on the level of depression, with women presenting with primary infertility (0.004) and those who had never conceive (0.001) tending to be more depressed (Table 2).

### Depression and age of the subjects

**Figure 1:**
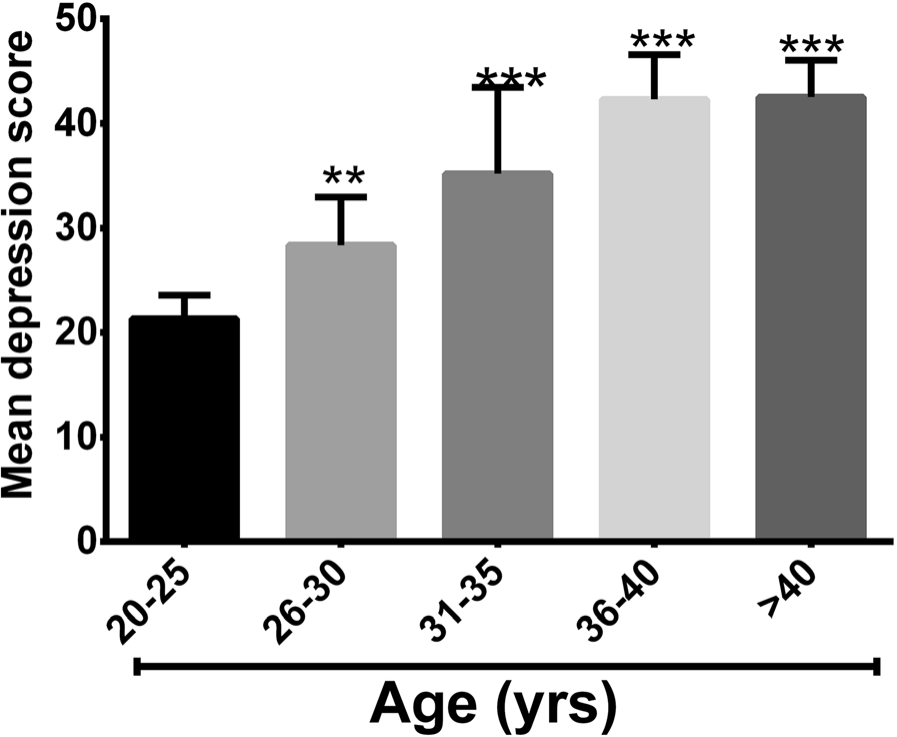
**Depression and age of the subjects.** Depression scores according to the age of the subjects. ** P <0.01, ***P < 0.001 compared to age group 20-25 (One-way ANOVA).

Figure 1 shows the mean depression score stratified by age of the respondents. Depression was more common in women age 26 years and above and highly significant in those women age 35 years and above (P < 0.001). The mean score of depression was higher in higher age until the age of 40 years and remain steady thereafter. There was a significant positive correlation between age of the subject and the BDI score for depression (r = 0.66, P < 0.0001, 95% CI = 0.4673 to 0.7926).

### Type of marriage and depression

An analysis of the type of marriage the study subjects were involved showed that, 52.0% and 48.0% of them were from Monogamous and Polygamous marriages respectively. The type of marriage did not have any significant influence on the mean score and level of depression (0.299) even though subjects from a polygamous marriage tended to experience depression than those from monogamous marriage (Table 1).

### Duration of infertility and depression

**Figure 2:**
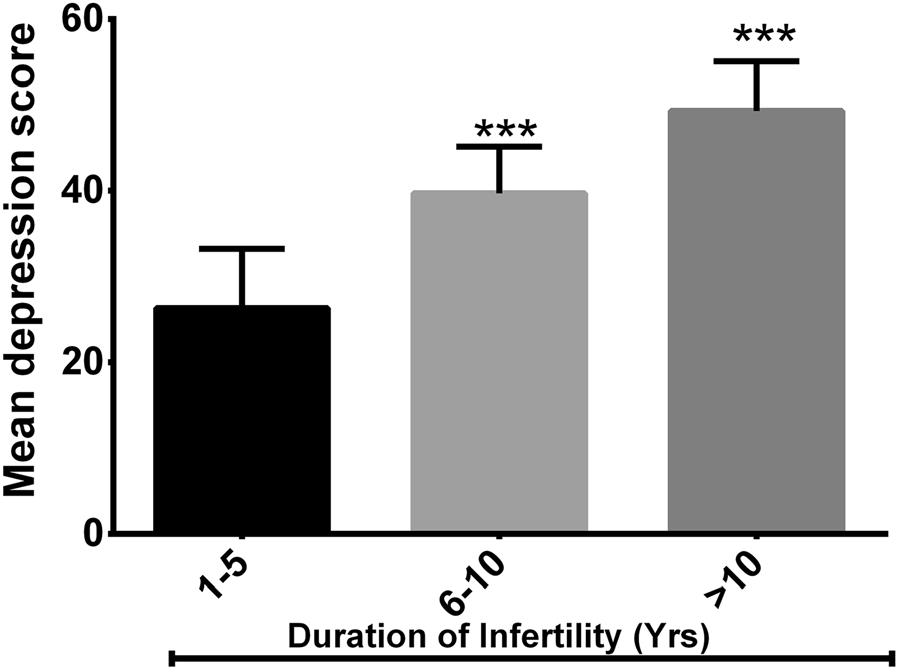
Duration of infertility and depression. ***P < 0.001 compared to 1-5 year group (One-way ANOVA). Depression score base on duration of infertility.

Stratified by duration of infertility 66%, 22% and 12% of the women had been suffering from infertility ranging from 1-5 years, 6-10 years, and >10 years respectively. Duration of infertility showed a significant position correlation with BDI score (r = 0.4736, P = 0.001, 95%CI = 0.216 to 0.667) (Figure 2).

## Discussion

The purpose of the present study was to determine the prevalence and severity of depression and its relationship to socio-demographic characteristics among a population of Ghanaian infertile women. In Ghana like other resource poor countries where children are highly valued for cultural, social and economic reasons, childlessness often creates huge problems for couples; especially for the women who are generally blamed for the infertility [14,22]. The stigma of childlessness is so great in Ghana that infertile women are socially isolated and neglected even by the people who are suppose to support them such as their husbands and extended family. Motherhood is often the only way for women to enhance their status within their family and community. These societal pressures may have some psychosocial impact on the infertile women in our society. To the best of our knowledge however this is the first cross-sectional study that has assessed depression among Ghanaian infertile women using the BDI. The finding of a high depression prevalence of 62.0% among the infertile women in the present study buttresses the psychological challenges that childless women are confronted with. This finding is consistent with the work of Guerra et al. [23] which also found about 67.0% depression among infertile women in China. The prevalence of depression in the present study is however higher than the findings of Ramezanzadeh et al. [19] which showed a prevalence of 40.8% among infertile women in Iran. The high prevalence of depression in the present study could be attributable to the societal and family demand on Ghanaian women to have their own children. In Ghana, children are seen as a form of social security in old age and as a means of perpetuating the family lineage. An additional reason could be due to the fact that this study was conducted in an area dominated by people practising the Islamic faith, where family status especially childbearing is very important and valuable. Here having children stabilizes family and increases marital satisfaction. Childless women therefore stand a risk of disrespectful treatment and stigmatization especially from relatives of the husband and may even lead to a divorce or another wife being brought into the marriage as permitted by Islamic law [19].

The mean depression score was high among women age 26 years and above and tended to be higher as the women advance in age. The mean score of depression however had a negative correlation with educational level as women who attained higher education had lower depression scores. Studies have shown that female fertility is at its peak around the ages of 26-35 years and infertility becomes more pronounced after the age of 35 [24,25]. The knowledge that their fertility may be declining after this age may put the women under some kind of psychological pressure which could have contributed to this high depression as noted in the present study. In most societies, higher education increases an individual’s chance of securing a well-paid and stable job which may relief the psychological impact of the infertility and this may explain the trend as observed in the present study with respect to mean depression score having a negative relation with educational and employment status. According to Ramezanzadeh et al. [19] housewives may experience more psychological signs of depression and anxiety more than those who work outside of the home. This finding is similar to the work of other researchers who have found a similar trend of depression with age as well as educational status [19,26].

Duration of infertility had a significant positive correlation with the mean BDI score (r = 0.4736, P = 0.001, 95%CI = 0.216 to 0.667). Women who had infertility duration of 3 years and above showed more depression. Several studies have also reported similar observations in different infertile populations [18,23,26,27]. During the early stages of being diagnosed with infertility the hopefulness of the woman for a successful outcome of medical intervention is higher. However, as the intervention progresses without a success combined with the stress of moving from one hospital to the other, they may become psychologically stress up with fading hopes of conception.

Previous studies have shown that primary infertility is not common in Ghana compare to secondary infertility [22]. This was in conformity with the present study where 62.0% of the women presented with secondary infertility. The type of infertility presented also had a significant influence on the mean depression score among the study subjects with women presenting with primary infertility having more psychological signs of depression. In primary infertility the woman has not been able to have a live birth putting into question her womanhood. This seemingly lost of identity as a woman is enough grounds for divorce in our society and can thus be a solid ground for serious psychological problems as shown in the present study.

## Conclusions

In conclusion, the prevalence of depression among the infertile women is high, especially among infertile women age 26 and above, those who are less educated, those with primary infertility, as well as those who have been diagnosed as infertile for more than 3 years. Interventions to decrease and prevent the development of severe depression among these patients should be considered.

## Competing interests

The authors declare that they have no competing interests.

## Authors’ contributions

AA developed the concept and designed the study. AA and ZA administered the questionnaire, analysed and interpreted the data. AA and ZA drafted the manuscript. AA, ZA and MS revised and copy-edited the manuscript for intellectual content. All authors read and approved the final manuscript.

## Pre-publication history

The pre-publication history for this paper can be accessed here:

http://www.biomedcentral.com/1472-6874/14/42/prepub
